# Supplementary figures and images for: Genetic variant in 3’ untranslated region of the mouse pycard gene regulates inflammasome activity
Source: eLife. 2021 Jul 1;10:e68203. doi: 10.7554/eLife.68203 (PMC8248980; doi:10.7554/eLife.68203)

Unedited western blots for Figure 5D. . A. ASC western blot. B. β-actin western blot.

A.


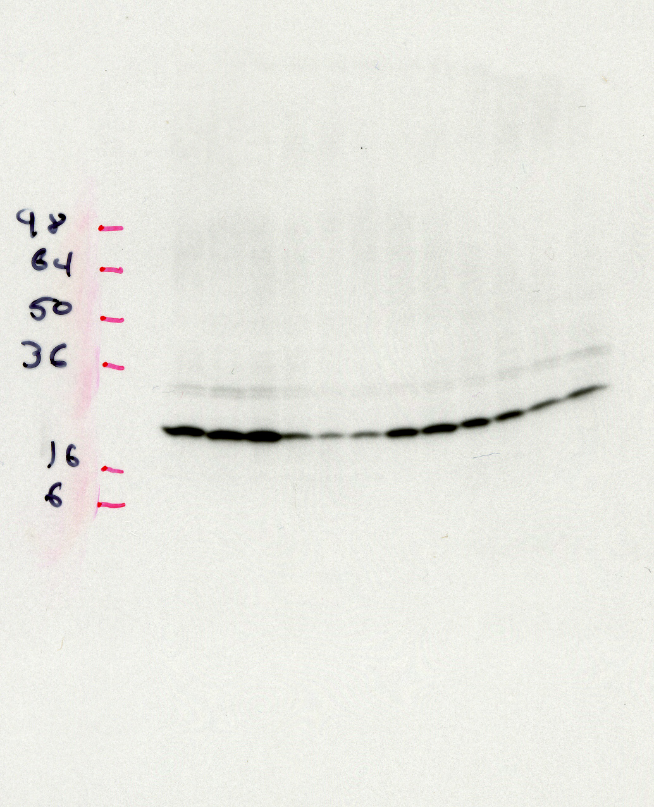


B.


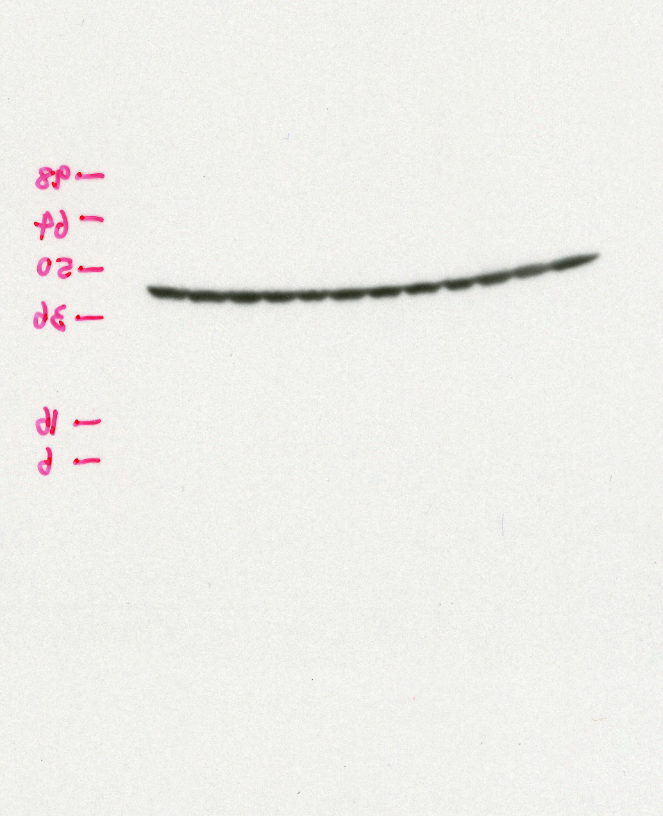

Supplement: Source data 1. — Unedited western blots in Figure 5D unedited western blot source data.docx. [file elife-68203-data1.zip › Figure 5D unedited western blot source data.docx]

Unedited western blots for Figure 2B. A. ASC western blot. B. β-actin western blot.

A.


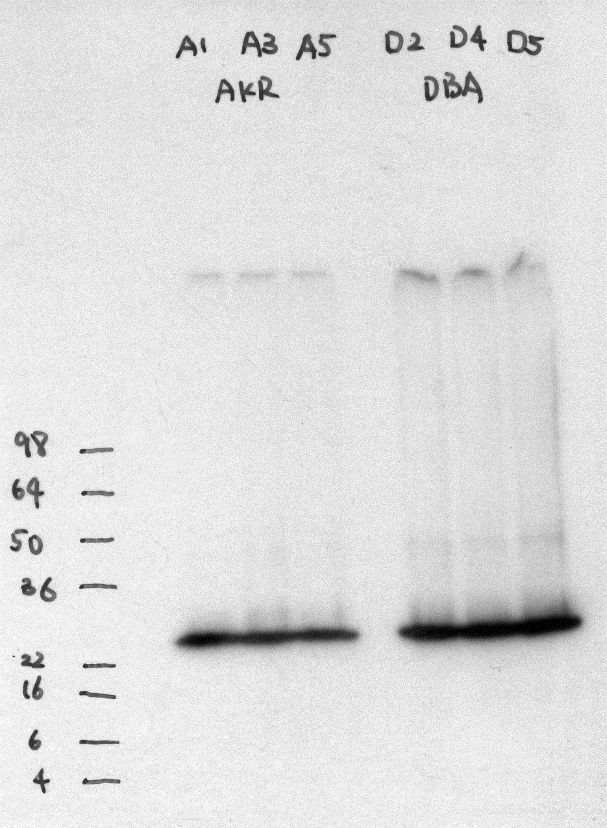


B.


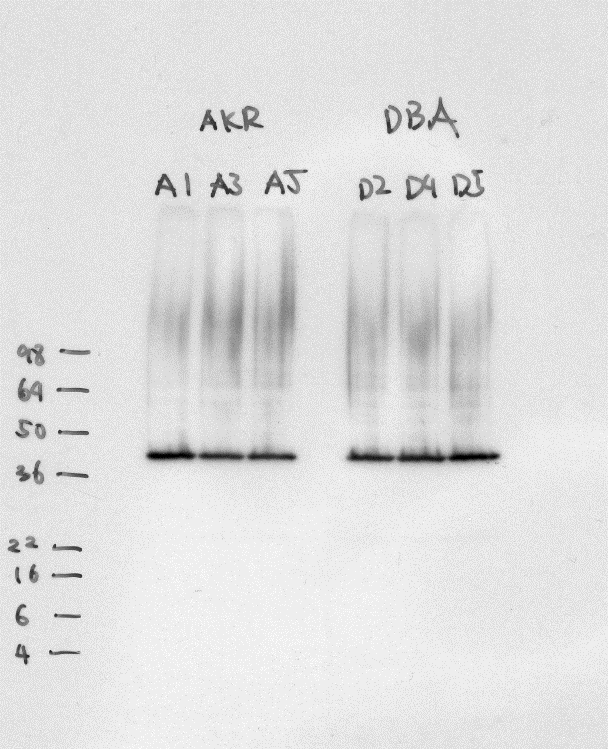

Supplement: Source data 1. — Unedited western blots in Figure 5D unedited western blot source data.docx. [file elife-68203-data1.zip › Figure 2B unedited western blot source data.docx]
