# Supplementary material for: Genetic variant in 3’ untranslated region of the mouse pycard gene regulates inflammasome activity
Source: eLife. 2021 Jul 1;10:e68203. doi: 10.7554/eLife.68203 (PMC8248980; doi:10.7554/eLife.68203)
Supplement: Supplementary file 1. — (a) Genes within the Irm3 QTL interval. All genes in the interval are shown along with the Mb position on chromosome 7. Other columns show distance from the LOD peak in Mb, whether a cis-eQTL was found and if so the LOD score for the eQTL, PubMed hits for the gene and the terns ‘inflammasome’ and ‘IL-1b’, the number of non-synonymous SNPs between the AKR and DBA/2 mouse strains, and the PROVEAN analysis to determine the number of non-synonymous SNPs likely to be deleterious to protein function. a, LOD score for cis-eQTL based on our prior BMDM strain intercross (Reference: J Hsu and JD Smith, PMID: 23525445 DOI: 10.1161/JAHA.112.005421); b, total number of PubMed hits for Boolean queries of the respective gene name and terms of interest.; c, non-synonymous SNPs between AKR and DBA/2 mice; d, PROVEAN, number of SNPs predicted to be deleterious by PROVEAN software. Yellow highlighting, top candidate gene. (b) Pycard gene sequencing PCR primer pairs. The sequence of the 6 PCR primer pairs used for Sanger sequencing of the Pycard gene in AKR and DBA/2 genomic DNA, along with the position of the primers relative to the transcription start site (TSS). [file elife-68203-supp1.docx]

**Supplementary Files**

**Supplementary File 1a. Genes within the *Irm3* QTL interval**

**Supplementary File 1b. *Pycard* gene sequencing PCR primer pairs**

Forward_1 GTCCCCATCCCTGCTTCCTCTCAC 4855 bp after TSS^a^

Reverse_1 CCAAACAGCCCTACGCATCTCCAG 3467 bp after TSS

Forward_2 GTGGGGCTTGAGACTGCTGGTGA 4063 bp after TSS

Reverse_2 TGGAGGGAATGAAGTTGATAGGTG 2836 bp after TSS

Forward_3 CCAGGGCTTGTATGTAGAGGTCA 3100 bp after TSS

Reverse_3 ATTTTGGGGGTGGGGCTGTTCATA 1806 bp after TSS

Forward_4 TATGAACAGCCCCACCCCCAAAAT 1829 bp after TSS

Reverse_4 GGCCTCCCCACCCTACCACACC 757 bp after TSS

Forward_5: GAAGCCTTTGCACTAGAATGGAGA 1387 bp after TSS

Reverse_5: ATGGGGCGGGCACGAGATG 213 bp after TSS

Forward_6: TGCGCCCATAGCCTTCTCG 329 bp after TSS

Reverse_6: AGCCTTAGCCCTTCCAACCCAACC 456 bp before TSS

^a^, TSS, transcription start site.
